# Efficacy and Safety of Varenicline for Smoking Cessation in Schizophrenia: A Meta-Analysis

**DOI:** 10.3389/fpsyt.2018.00428

**Published:** 2018-09-19

**Authors:** Saeed Ahmed, Sanya Virani, Vijaya P. Kotapati, Ramya Bachu, Mahwish Adnan, Ali M. Khan, Aarij Zubair, Gulshan Begum, Jeevan Kumar, Mustafa Qureshi, Rizwan Ahmed

**Affiliations:** ^1^Nassau University Medical Center, East Meadow, NY, United States; ^2^Maimonides Medical Center, New York, NY, United States; ^3^Manhattan Psychiatric Center, New York, NY, United States; ^4^Zucker Hillside Hospital, New York, NY, United States; ^5^Department of Cognitive Behavioral Science, McMaster University, Hamilton, ON, Canada; ^6^Department of Psychiatry, University of Texas Rio Grande Valley, Harlingen, TX, United States; ^7^St. John's University, New York, NY, United States; ^8^Bolan Medical College, Quetta, Pakistan; ^9^Texas Tech University Health Sciences Center, Lubbock, TX, United States; ^10^Liaquat National Medical College, Karachi, Pakistan

**Keywords:** schizophrenia, cigarettes, nicotine addiction, smoking cessation, varenicline

## Abstract

**Objective:** Smoking represents a major public health problem among patients with schizophrenia. To this end, some studies have investigated the efficacy of varenicline for facilitating smoking cessation in schizophrenia patients. The present review seeks to synthesize the results of these studies as well as document the reported side effects of using this medication.

**Methods:** An electronic search was performed using five major databases: PubMed, Scopus, EMBASE, Web of Science, and Cochrane Library. Included in the current analysis were randomized clinical trials (RCTs) that have investigated the effect of varenicline in promoting smoking cessation in patients with schizophrenia. Risk of bias among included RCTs was assessed using the Cochrane Collaboration's quality assessment tool.

**Results:** Among the 828 screened articles, only four RCTs, which involved 239 participants, were eligible for meta-analysis. In patients with schizophrenia, varenicline treatment when compared to placebo significantly reduced the number of cigarettes consumed per day [SMD (95% CI) = 0.89(0.57–1.22)] and expired carbon monoxide levels [SMD (95% CI) = 0.50 (0.06–0.94)] respectively.

**Conclusion:** Despite a limited number of studies included in the meta-analysis, our results suggest that varenicline is an effective and safe drug to assist smoking cessation in patients with schizophrenia. Future large-scale well-designed RCTs are required to validate these findings.

## Introduction

Smoking among patients with schizophrenia continues to be a major public health problem. There is a strong association between schizophrenia and smoking behaviors, with prevalence rates ranging from 70 to 90%; as a comparison, smoking rates of the general population in the United States stands at ~30% (1, 2). Also, compared to smokers in the general population, schizophrenic smokers consume more cigarettes per day (3). Schizophrenic patients usually smoke heavily, extract higher levels of nicotine, and have higher nicotine dependence scores than smokers who do not have schizophrenia (3–7).

Several hypotheses have been proposed to explain the high rates of smoking in this patient population. The most popular hypothesis, which is known as “self-medication hypothesis (SMH)”, argues that these patients smoke in order to manage their negative symptoms by regulating and compensating for the underlying neurobiological deficits associated with the disorder such as cognitive deficits. Smoking may transiently alleviate negative symptoms in schizophrenic patients by increasing dopaminergic and glutamatergic neurotransmission in the prefrontal cortex. Also, nicotine improves some cognitive deficits common in patients with schizophrenia, such as sensory gating deficits, abnormalities in smooth-pursuit eye movements, selective attention, visuospatial working memory limits, and attention deficits; also, high-dose nicotine increases abnormality in P450 inhibition (8). Contrary to these temporary benefits, there is abundant evidence that clearly shows that smoking is harmful because it increases the risk of contracting numerous medical illnesses such as lung cancer and other smoking-related diseases. Mortality rates also reflect this trend; patients with schizophrenia who smoke face mortality rates from smoking-related health issues that are two to six times higher than other smokers. They confront increased risks of cardiovascular disease, respiratory disease, and cancer (9). The increased mortality risks that result from smoking exacerbates already high mortality risk schizophrenic patients. This higher mortality risk results in about a 20–25% reduction in average lifespan for people with schizophrenia (4). Schizophrenic patients are at higher risk of fatal cardiovascular disease and other causes of premature death such as obesity, sedentary lifestyle, diabetes, hypertension, or hypertriglyceridemia (10). The risk of HIV (human immunodeficiency virus) and infectious hepatitis is also high in this population. The suicide rate is 10% compared to 1% in the general population; the most likely explanations for this higher risk of death include less access to medical care, engagement in high-risk behaviors, and poorer compliance with treatment (11). Concerning morbidity in particular, second-generation antipsychotics are more likely to cause weight gain and metabolic syndrome compared to first-generation antipsychotics, and these side effects are associated with a two-to-threefold increase in cardiovascular mortality and a twofold increase in mortality in general (12). The increased risk of mortality in schizophrenics affects men and women equally, although there are sex differences in the epidemiological features of schizophrenia (12). Therefore, the added health risks associated with smoking are particularly dangerous to this population and must be addressed.

Clinicians often do not address nicotine addiction in the schizophrenic population because of the popular beliefs that treatment could worsen the patient's illness or that the patient is not willing to quit (3). However, numerous studies have demonstrated that stable patients with schizophrenia can tolerate cessation attempts without an overall worsening of their illness and can have moderate short-term success in smoking cessation (2–4, 13–22). Once these patients demonstrate a willingness to quit smoking, they can try numerous therapeutic options that have proven to be beneficial. In 2006, the FDA approved varenicline, a partial agonist of α4β2 acetylcholine receptor and a full α7 nicotinic acetylcholine receptor agonist for smoking cessation. This drug ameliorates withdrawal symptoms, specifically reducing cravings, and it works as a positive reinforcement for the cessation of smoking. However, there have been some reports of side effects that include the exacerbation of psychiatric symptoms such as depression, aggression, psychosis, and suicidal thoughts and behaviors (23–28). As a result of these sometimes dangerous side effects, the FDA issued a black box warning in 2009 (29). However, several recent studies have provided evidence regarding its safety in facilitating smoking cessation in patients with schizophrenia (9, 15, 30–33). Numerous published clinical trials have found varenicline to be effective, safe, and well tolerated in terms of neuropsychiatric adverse events, with a common possible side effect of mild to moderate nausea similar to that experienced by the general populations (3, 4, 34–39). A recently published meta-analysis of this subject concluded that varenicline is not superior to placebo in smoking cessation (40). However, the results of this metanalysis run counter to numerous studies that have found varenicline to be an effective, safe, and well-tolerated treatment for smoking cessation in schizophrenic patients (3, 33, 41, 42). Because of these positive findings as well as a new study on this topic (43), we sought to synthesize previous findings and examine the overall effect of varenicline on smoking cessation among patients with schizophrenia.

## Methods

### Search strategy and selection criteria

The Recommendations from the Preferred Reporting Items for Systematic Reviews and Meta-Analyses (PRISMA) Statement were adopted to conduct this systematic review and meta-analysis (44). Five databases were systematically searched including PubMed, Scopus, EMBASE, Web of Science and Cochrane's library for articles published up until July 2018. We performed an electronic search on this topic with no restrictions regarding language or publication date. The Boolean search we conducted utilized the following keyword combinations: varenicline AND [(schizophrenia spectrum disorder) OR schizophrenia OR (schizoaffective disorder) OR (schizophreniform disorder) OR (delusional disorder)], the strategy was adjusted to suit each of the chosen search engines and databases. We further considered performing a manual search in reference lists of relevant studies. Once we completed the search, we elected to include only RCTs that investigated the effects of varenicline on either smoking cessation or smoking reduction in patients with schizophrenia, schizophreniform, schizoaffective, or delusional disorder. We excluded reviews, case series, case reports, comments, opinion, unpublished studies, conference posters, and abstracts only. We also excluded studies with overlapped data or data that could not be extracted. Three authors (SA, PVK, and RA) blindly screened all articles obtained through the search, based on titles and abstracts, to identify relevant articles for full-text consideration. Discrepancies were resolved by discussion, and consensus was achieved. Table 1 shows the list of studies considered in the final stage of selection, the decision about whether or not to include each relevant study, and the reasons for excluding the articles which were not chosen.

**Table 1:** List of studies considered at the final stage of study selection.

**Study**	**Included?**	**Reason for exclusion**
Dutra et al. (42)	No	No control group
Williams et al. (33)	Yes	
Pachas et al. (9)	No	No control group
Fatemi et al. (45) (clinicaltrial.gov)	No	No baseline measures with which to assess outcomes
Weiner et al. (19)	Yes	
Jeon et al. (43)	Yes	
Coles et al. (32) (poster abstract)	No	No relevant data to extract. Conference poster; only abstract available
Hong et al. (46)	Yes	

### Data extraction

Data were extracted by three blinded authors in order to preserve precision, using a template in Microsoft Excel to extract the needed data. The data that was extracted included sample size, age of subject, sex of subject, follow-up period, and drug dosage. Additionally, data for tested outcomes, the number of cigarettes smoked per day, expired carbon monoxide, and abstinence from smoking behavior were extracted and meta-analyzed.

### Statistical methods

For dichotomous outcomes which are determined only at post-treatment, (such as the number of participants who remained abstinent), the log Odds Ratios (OR) and their respective sampling variances were calculated using the escalc function in the meta for package (47) in R. As for continuous outcomes studied both at baseline and post-treatment, the raw score, single group, pretest-posttest standardized mean difference (SMD) for both the treatment and placebo groups were computed separately (48). Their respective sampling variances were computed using formula A1 in (48). After this computation was complete, the placebo group's SMD was subtracted from that of the treatment group to obtain the overall SMD for a researched outcome in the study. The sampling variances of the treatment and placebo subgroups were then added together to obtain the overall sampling variance for that outcome in the study (48).

These SMDs were then pooled and meta-analyzed using random effects models (REM) to allow the effect sizes to vary across studies. The direction of the effect sizes was coded in such a way that larger positive effect sizes corresponded to larger positive differences (i.e., treatment–placebo) in raw scores. Heterogeneity was measured using the Q statistic; a significant Q statistic suggests that the variability among the effect sizes is larger than what is expected from subject sampling error alone. Follow-up leave-one-out analyses were carried out to assess the robustness of the results. These analyses were performed using the metafor package (47) in R 3.4.0.

### Quality assessment

The included studies were independently assessed using Cochrane collaboration's tool for assessing risk of bias (49). This tool is a two-part tool that addresses seven specific domains, including sequence generation, allocation concealment, blinding of participants, and personnel, blinding of outcome assessment, incomplete outcome data, selective outcome reporting, and other sources of bias. The judgment of each reviewer of each domain was categorized as “low risk,” “high risk,” or “unclear risk” of bias. Any disagreement was resolved by discussions between reviewers until a consensus was reached.

## Results

### Search results

**Figure 1:**
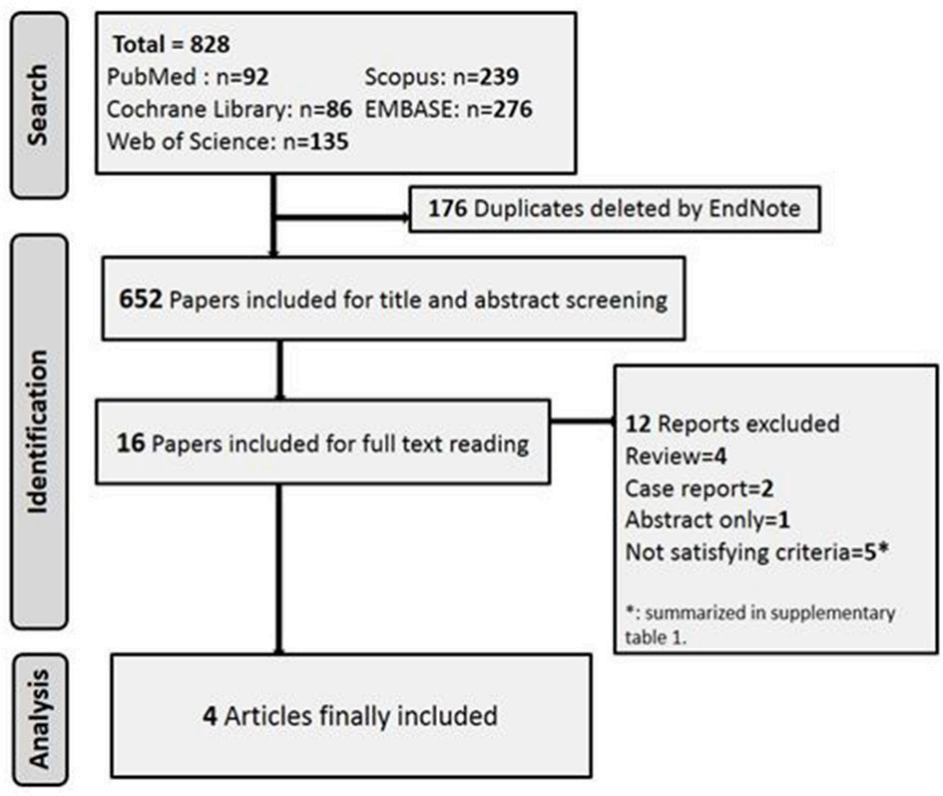
PRISMA flow diagram of studies' selection and screening.

The literature search yielded 822 articles. Five RCTs investigated the efficacy of varenicline in different clinical conditions and thus did not fulfill the core requirements of this meta-analysis. We summarize their interventions and the reasons for exclusion of these studies in Table 1 (9, 32, 42, 45). The flowchart of studies' selection and screening is presented in Figure 1.

### Baseline characteristics

Our four included RCTs were comprised of 239 participants at baseline; however, only 191 completed the study and follow-up. The DSM-IV confirmed only schizophrenia in only one study (43), while schizophrenia or schizoaffective disorder was present in the participants in the remaining studies (19, 33, 46). The participants' mean age ranged from 40 to 43, and most of patients were male. In one of the studies, researchers did not indicate the age or gender of included participants (19). The treatment duration was 12 weeks in two studies (19, 33) and only 8 weeks in the two remaining (43, 46). The characteristics of the studies are detailed in Table 2.

**Table 2:** Characteristics of included studies.

**References**	**DSM-IV diagnosis**	**Treatment duration in weeks**	**Dose (mg)**	**Sample size**				**Age mean (*****SD*****)**		**Male (%)**	
				**Baseline**		**Follow-up**		
				**Varenicline**	**Placebo**	**Varenicline**	**Placebo**	**Varenicline**	**Placebo**	**Varenicline**	**Placebo**
Williams et al. (33)	SCZ/SCA	12	0.5 × 3days, 1 × 4days, 2/day thereafter	84	43	61	37	40.2 (11.9)	43 (10.2)	77.4	76.7
Jeon et al. (43)	SCZ	8	0.5 × 3days, 1 × 4days, 2/day thereafter	30	30	23	22	41.1 (8.9)	41.5 (8.8)	86.7	96.7
Hong et al. (46)	SCZ/SCA	8	0.5 × 1week, 1 × 7week	20	23	19	21	43 (11)	41.5 (11.4)	65	60.9
Weiner et al. (19)	SCZ/SCA	12	1/day	4	5	4	4	ND	ND	ND	ND

*SD, standard deviation; SCZ, schizophrenia; SCA, schizoaffective; d, days*.

### Risk of bias among included RCTs

**Figure 2:**
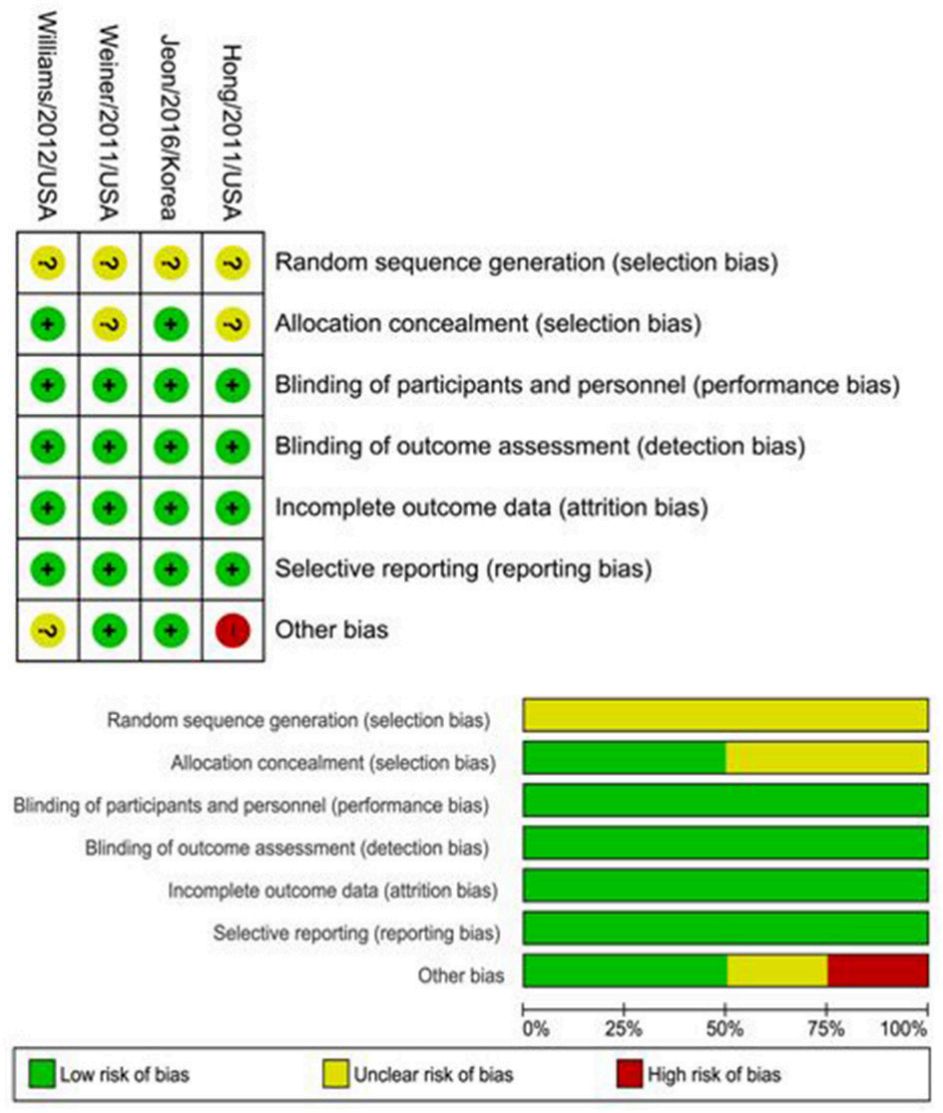
Summary of risk of bias assessment according to Cochrane collaboration tool.

The random sequence generation presented an unclear risk of bias among the four included RCTs. Performance, detection, attrition, and reporting bias were low in all studies. Regarding allocation concealment, there was an unclear risk of bias in two studies (19, 46) while there was only a low risk in the remaining two (33, 43). The risk of bias in the studies is characterized in Figure 2.

### Outcomes

#### Impact of varenicline on the cigarette consumption in schizophrenia patients

**Figure 3:**
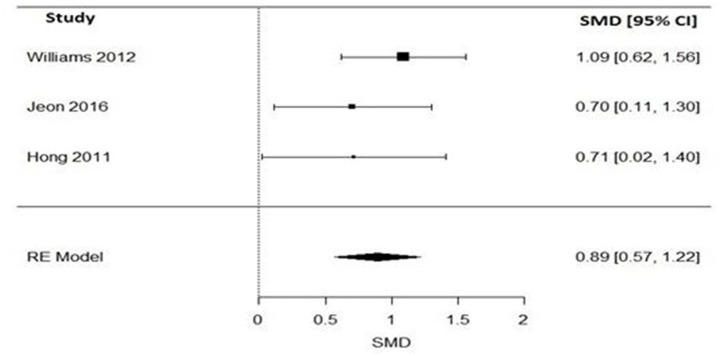
Forest plot meta-analysis of three RCTs illustrating impact of varenicline compared to placebo on cigarette consumption in schizophrenia patients. SMD, standard mean difference; CI, confidence interval; RE, random effect.

Pooling together the three studies that evaluated the impact of varenicline on the number of cigarettes consumed per day, schizophrenic smokers who were given varenicline (*N* = 134) had significantly reduced the number of cigarettes consumed per day, when compared to those given a placebo (*N* = 94) (Figure 3). The adverse events among included RCTs are shown in Table 4. The Q statistic did not suggest significant heterogeneity in the random effects model (Table 5). Additionally, the leave-one-out analysis suggested that the obtained estimate was generally robust and that removal of any of the three studies did not alter the statistical significance (Table 3).

**Table 3:** Summary of leave-one-out analysis.

**Study removed**	**SMD**	**95% CI**	**Q**
Williams et al. (33)	0.71^**^	0.26–1.16	<0.001
Jeon et al. (43)	0.97^***^	0.59–1.36	0.79
Hong et al. (46)	0.94^***^	0.57–1.31	1.01

SMD, standardized mean difference; CI, confidence intervals;

** p < 0.01;

*** *p < 0.001*.

**Table 4:** Adverse events among included RCTs.

**References**	**Groups**	**GIT manifestation N (%)**					**Psychiatric disorders N (%)**					**General condition N (%)**	
		**Nausea**	**Diarrhea**	**Constipation**	**Vomiting**	**Abdominal pain**	**Abnormal dreams**	**Anxiety**	**Suicidal ideation**	**Insomnia**	**Depression**	**Headache**	**Fatigue**
Williams et al. (33)	Varenicline	20 (23.8)	7 (8.3)	ND	9 (10.7)	7 (8.3)	6 (7.1)	4 (4.8)	5 (6)	8 (9.5)	4 (4.8)	9 (10.7)	5 (6)
	Placebo	6 (14)	2 (4.7)	ND	4 (9.3)	1 (2.3)	4 (9.3)	4 (9.3)	3 (7)	2 (4.7)	3 (7)	8 (18.6)	2 (4.7)
Hong et al. (46)	Varenicline	10 (31.3)	ND	5 (15.6)	5 (15.6)	9 (28.1)	3 (9.4)	7 (21.9)	ND	9 (28.1)	ND	3 (9.4)	13 (40.6)
	Placebo	10 (31.3)	ND	8 (25)	1 (3.1)	11 (34.4)	11 (34.4)	6 (18.8)	ND	11 (34.4)	ND	10 (31.3)	10 (31.3)
Weiner et al. (19)	Varenicline	3 (75)	ND	2 (50)	ND	ND	ND	ND	ND	3 (75)	ND	ND	ND
	Placebo	1 (20)	ND	0	ND	ND	ND	ND	ND	1 (20)	ND	ND	ND

*ND, not detected*.

**Table 5:** Analysis summary of the parameters studied and their outcome.

**Outcome**	**Varenicline N**	**Placebo N**	**Estimate**		**95% CI**	**Q**
			**Type**	**Effect**		
Cigarettes/day	133	94	SMD	0.89^***^	0.57, 1.22	1.33
Expired carbon monoxide	49	51	SMD	0.50^*^	0.06, 0.94	0.22
Abstinence	88	47	Log OR	1.81^*^	0.41, 3.20	0.57

N, number; SMD, standardized mean difference; OR, Odds Ratio; CI, confidence intervals;

* p < 0.05;

*** *p < 0.001*.

#### Impact of varenicline on the expired carbon monoxide in schizophrenia patients

**Figure 4:**
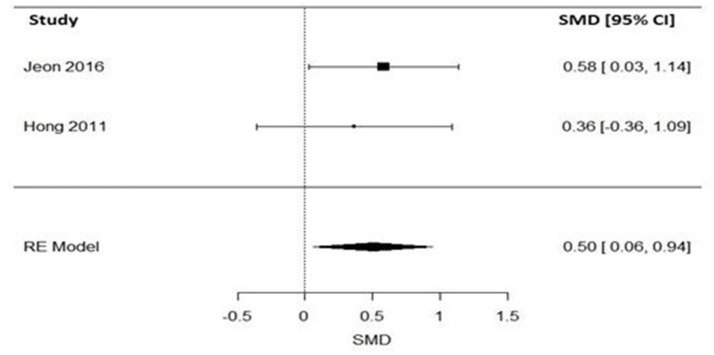
Forest plot meta-analysis of two RCTs for the effect of varenicline compared to placebo on the expired carbon monoxide in schizophrenia patients. SMD, standard mean difference; CI, confidence interval; RE, random effect.

Regarding levels of carbon monoxide, only two studies evaluated the impact of varenicline on the expired carbon monoxide levels before and after treatment (43, 46). Varenicline treatment (*N* = 49), compared to placebo (*N* = 51) significantly reduced the expired carbon monoxide levels in schizophrenia patients (Figure 4). Similarly, the Q statistic did not indicate significant heterogeneity in the random effects model (Table 5).

#### Impact of varenicline on the abstaining from smoking behavior in schizophrenia patients

**Figure 5:**
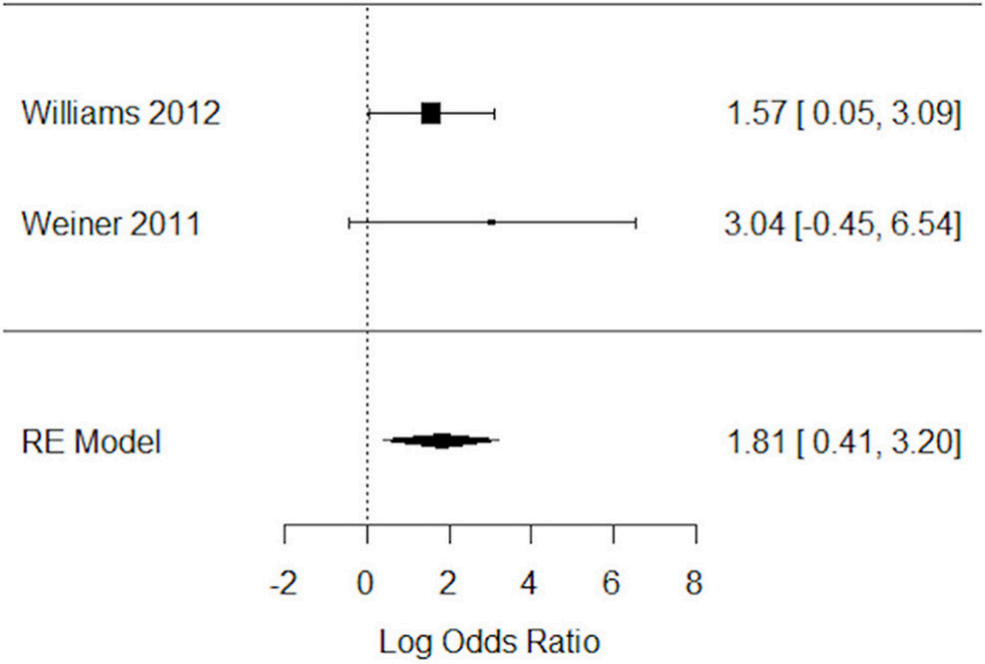
Forest plot meta-analysis of two RCTs for the impact of varenicline on abstaining from smoking behavior in schizophrenia patients. CI, confidence interval; RE, random effect.

Two studies have assessed the impact of varenicline on abstinence rates before and after treatment (19, 33). Again, varenicline-treated schizophrenic patients (*N* = 87), relative to placebo group (*N* = 47), had significantly higher rates of abstaining from smoking behavior (Figure 5). The Q statistic did not indicate significant heterogeneity in the random effects model (Table 5).

#### Adverse events of varenicline compared to placebo in schizophrenic patients

The side effects reported in the included RCTs included gastrointestinal and psychiatric symptoms, as well as headache and fatigue. Nausea and insomnia were reported in three RCTs (19, 33, 46), while vomiting, abdominal pain, abnormal dreams, anxiety, headache, and fatigue were reported in two studies (33, 46). In only one study did some patients experience diarrhea and depression (33). Detailed information on reported adverse events are displayed in Table 4.

## Discussion

Varenicline has not been studied in patients with mental illnesses exclusively, particularly in patients with schizophrenia, a population with an exceptionally high prevalence of nicotine addiction. The safety and tolerability of varenicline has been debated and questioned, especially after initial case reports and small case series showed an increased risk of neuropsychiatric adverse effects (23, 24, 50–53) in psychiatric populations. However, subsequent studies from case reports and case series to larger clinical trials (9, 15, 19, 31, 33, 37, 42, 43, 45, 46, 53–59) have demonstrated that the medication is well tolerated and safe to use to aid schizophrenia patients with smoking cessation.

Though the therapeutic potential of varenicline has been questioned in a recent meta-analysis (40), a thorough review of published studies in recent years provides adequate evidence that varenicline is an effective aid in smoking cessation in the schizophrenic population. A systematic review of the Cochrane database was conducted by (41) to evaluate the benefits and drawbacks of different treatments for nicotine dependence in schizophrenia. The authors found that smokers with schizophrenia who used varenicline were nearly five times more likely to abstain from smoking at the end of the treatment when compared to the placebo group. However, these results were obtained by analyzing a very small sample consisting of only two trials (19, 33) (2 trials *N* = 137, RR 4.74, 95%CI 1.34 to 16.71).

Looking at more evidence from published trials such as Weiner et al. (19) studied varenicline for smoking cessation in eight patients with schizophrenia. In this RCT, the authors attempted to test the safety, tolerability, and efficacy of varenicline. The study determined varenicline to be safe and well-tolerated by schizophrenic patients (three of the four participants in the varenicline group were able to achieve sustained abstinence) and were not associated with any worsening of negative symptoms or other psychiatric symptoms as measured using BPRS scales. Overall, the side effects reported in the varenicline group included nausea, insomnia, constipation, all of which have been reported as side effects in the general population as well. Similarly, a 12-week randomized trial by William et al. (33) studied the efficacy of varenicline for smoking cessation in patients with stable schizophrenia or schizoaffective disorder. The study reported that at the end of treatment, 16 out of 84 in the varenicline group (19.0%) met smoking-cessation criteria vs. 2 out of 43 (4.7%) for the placebo group (*P* = 0.046). The study found varenicline to be a promising aid for smoking cessation, with higher rates of significance than placebo. Additionally, the study indicated that varenicline was well tolerated in schizophrenic patients for smoking cessation and did not show any evidence of exacerbation of neuropsychiatric symptoms,

In 2015, a meta-analysis was conducted by Roberts et al. to assess the efficacy and tolerability of adjunctive pharmacotherapy for smoking cessation in patients with severe mental illnesses including schizophrenia, schizoaffective disorder, bipolar disorder, delusional disorder, and depressive psychoses (60). The authors analyzed published studies to examine the various smoking cessation pharmacotherapy modalities including nicotine replacement therapy (NRT), bupropion, and varenicline. The meta-analysis of a total of 17 studies suggested that bupropion and varenicline were more effective than placebo in a severely mentally ill population. A year later, another meta-analysis was conducted by Wu et al. (61) to determine the safety and effectiveness of varenicline in treating tobacco dependence in patients with severe mental illness, one of which was schizophrenia. This review analyzed eight randomized controlled trials in which the researchers compared varenicline with placebo or an alternative intervention for smoking cessation or reduction. The primary outcome measures of interest in the study were smoking cessation, measured by observing a change in the number of cigarettes smoked per day and the safety of varenicline smoked per day and the safety of varenicline, measured by noting the number of adverse psychiatric events. The study demonstrated that varenicline was significantly more effective than placebo, increasing the chance of success fourfold when compared to placebo; also, the reduction in smoking was greater in people with severe mental illness (SMI) when compared to the placebo group. The study defines SMI broadly to include non-organic disorders with psychotic features, schizophreniform and schizoaffective disorders, bipolar disorder, or delusional disorder. Although the study's results were found in favor of varenicline, the major limitation of this analysis was its inclusion of small-size trials, and the follow-up periods in all of the studies were short.

Kishi et al. (40) conducted a meta-analysis of randomized, double-blind, placebo-controlled trials (RCTs) to find the effects of varenicline adjuvant therapy for smoking cessation in people with schizophrenia. The results of the study suggested that, varenicline adjuvant therapy is not superior to placebo for smoking cessation in people with schizophrenia. Further, the study found that varenicline adjuvant therapy failed to show its superiority to placebo for positive, negative, and depressive symptoms. However, the study concluded that varenicline is well tolerated for smoking cessation in this population. Because of the many limitations of their meta-analysis including small-sample-size studies; the authors suggest further studies to examine varenicline on a larger scale with larger sample sizes. Nevertheless, the results of this meta-analysis came as a surprise to the psychiatric community, especially addiction psychiatrists. As a result of this study, Evins et al. (62) responded to this meta-analysis with a letter that highlighted the errors of the meta-analysis and mentioned that Kishi et al. (40) inappropriately included two trials which were not smoking cessation trials and that those trials were conducted on schizophrenic patients who had no intention of quitting. This caused the investigators to draw the erroneous conclusion that varenicline is ineffective for smoking cessation. Additionally, the meta-analysis inappropriately included a RTC trial by Shim et al. in which varenicline was examined as a cognitive enhancing agent (57). The meta-analysis also inappropriately included a relapse prevention trial of smoking cessation in schizophrenia patients (63). Although it is highly commendable that Kishi et al. (40) examined the effectiveness and safety of varenicline, a traditionally understudied topic. Because of analysis included inappropriate trial studies, the results of meta-analysis were opposite to numerous evidenced-based trials. Such error could lead readers to draw the wrong conclusions; therefore, the results could cause potential harm and lead to an underuse of this medication in this population.

Such erroneous findings as discussed above urged scientific community including authors of the current study to conduct an updated meta-analysis. Consequently, we did the meta-analysis using comprehensively defined inclusion and exclusion criteria. The results of our study show that varenicline is an effective and safe drug to use for smoking cessation in patients with schizophrenia. It is well tolerated and an effective aid in reducing cigarette consumption. The novelty of the current study is evidenced by results of the study, which were drawn after modifying and refining our analytic methodology. We expanded the parameters literature search, added recently published studies especially after 2015. Further, our study has a few strengths such as refining our inclusion criteria to target only studies that elucidated the efficacy and safety of varenicline for smoking cessation in schizophrenic patients. We did not consider any relapse prevention studies nor trials that examined the role of varenicline as a cognition-enhancing drug for smokers and nonsmokers; such an addition might have contaminated the sample and therefore lead to erroneous results. We assessed the quality of evidence by using the Cochrane tool, which is the universally accepted and recommended methodology for analysis of randomized trials. Moreover, compared to previously published meta-analysis, our electronic search for studies was carried out on five major databases, PubMed, Scopus, EMBASE, Web of Science, and Cochrane Library, a reasonably appropriate large search for performing a systematic review and meta-analysis.

Our study has suffered from limitations such as the small number of included participants; thus, our results should be implemented cautiously in clinical practice. The studies which we didn't include in the study are Dutra et al. (42), Pachas et al. (9), Fatemi et al. (2), Coles et al. (32) due to the lack of common variables and measures to assess final outcomes and the lack of relevant data from which we could extract information such as conference posters or abstracts only. Additionally, we excluded studies for which there was no control group (Table 1). Another limitation of our study was the unclear risk of bias regarding the selection of participants among all included RCTs. We also could not carry out subgroup meta-analysis or meta-regression due to the small number of included RCTs. We did not conduct pre- and post-meta-analysis for studies with no control group, and only those with a control arm were eligible for inclusion within our study.

## Conclusion and future perspectives

While the current meta-analysis demonstrates therapeutic efficacy of varenicline in promoting smoking cessation in clinically stable schizophrenic patients, however the concussion is subjected to certain limitations. Because of our well-established inclusion criteria and because of the relatively few clinical studies performed on this topic, we analyzed only a small number of studies. However, larger controlled trials on this topic are needed to draw firm and evidence based conclusion. Future clinical studies should consider assessing the impact of the duration of varenicline treatment, its role in relapse prevention over extended periods of time, dose dependent responses for smoking cessation, and consider smoking reduction in addition to smoking cessation.

## Author contributions

SA, SV, VK, and MA: Study conception. SA, RA, AZ, SV, and JK: Study design. RB, AK, MQ, SA, and JK: Data acquisition. SA, AK, RA, AZ, MQ, and MA: Data analysis and interpretation. SA, SV, AK, MQ, and RB: Writing the paper draft. SA, MQ, RA, AZ, RB, and MA: Critical revision of the manuscript. GB: Interpretation, writing the paper draft, critical revision of the manuscript.

### Conflict of interest statement

The authors declare that the research was conducted in the absence of any commercial or financial relationships that could be construed as a potential conflict of interest.
